# 4′-Cyano­biphenyl-4-yl 7-diethyl­amino-2-oxo-2*H*-chromene-3-carboxyl­ate

**DOI:** 10.1107/S1600536813001591

**Published:** 2013-01-19

**Authors:** S. Sreenivasa, H. T. Srinivasa, B. S. Palakshamurthy, Vijith Kumar, H. C. Devarajegowda

**Affiliations:** a Deapartment of Studies and Research in Chemistry, Tumkur University, Tumkur 572 103, Karnataka, India; b Raman Research Institute, C. V. Raman Avenue, Sadashivanagar, Bangalore 560 080, Karnataka, India; c Department of Physics, Yuvaraja’s College (Constituent College), University of Mysore, Mysore 570 005, Karnataka, India; d Soild State and Structural Chemistry Unit, Indian Institute of Science, Bangalore 560 012, Karnataka, India

## Abstract

In the title compound, C_27_H_22_N_2_O_4_, the dihedral angles between the central benzene ring and the cyano­benzene ring and the 2*H*-coumarin ring system (r.m.s. deviation = 0.014 Å) are 22.95 (11) and 75.59 (8)°, respectively. Both terminal C atoms of the pendant diethyl­amino group lie to the same side of the coumarin ring system [deviations = 1.366 (2) and 1.266 (2) Å]. In the crystal, mol­ecules are linked by C—H⋯O and C—H⋯N hydrogen bonds and a C—H⋯π inter­action, generating a three-dimensional network.

## Related literature

For the biological properties of coumarin derivatives, see: Bhat *et al.* (2006 ▶); Chimichi *et al.* (2002 ▶).
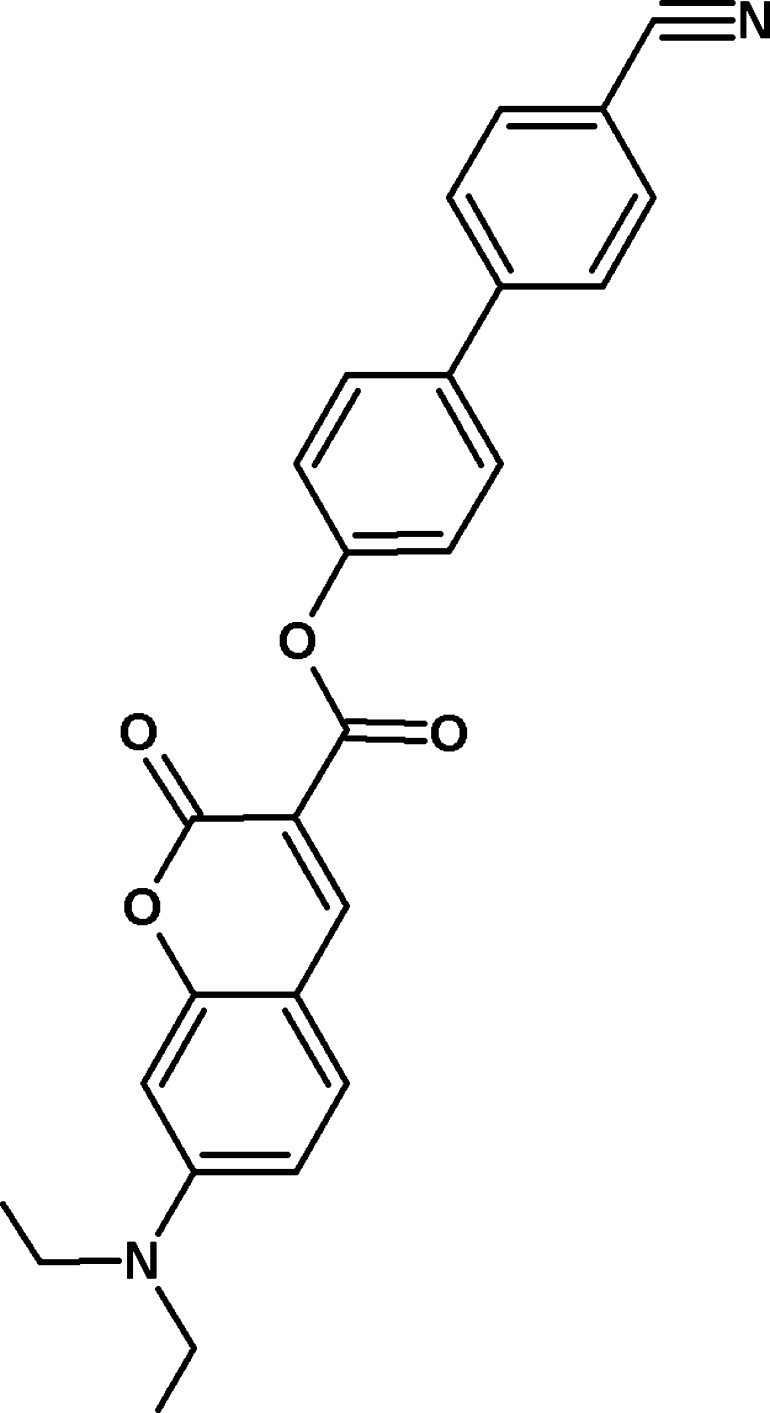


## Experimental

### 

#### Crystal data

C_27_H_22_N_2_O_4_
*M* *_r_* = 438.47
Triclinic, 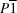

*a* = 9.652 (3) Å
*b* = 10.252 (4) Å
*c* = 11.121 (4) Å
α = 87.214 (10)°
β = 86.358 (10)°
γ = 84.348 (11)°
*V* = 1091.9 (6) Å^3^
*Z* = 2
Mo *K*α radiation
μ = 0.09 mm^−1^
*T* = 300 K
0.24 × 0.20 × 0.18 mm

#### Data collection

Bruker SMART CCD diffractometer
Absorption correction: multi-scan (*SADABS*; Bruker, 2001 ▶) *T*_min_ = 0.979, *T*_max_ = 0.984
10667 measured reflections
3783 independent reflections
2495 reflections with *I* > 2σ(*I*)
*R*_int_ = 0.048

#### Refinement

*R*[*F* ^2^ > 2σ(*F* ^2^)] = 0.058
*wR*(*F* ^2^) = 0.173
*S* = 0.97
3783 reflections
301 parameters
H-atom parameters constrained
Δρ_max_ = 0.22 e Å^−3^
Δρ_min_ = −0.37 e Å^−3^

### 

Data collection: *SMART* (Bruker, 2001 ▶); cell refinement: *SAINT* (Bruker, 2001 ▶); data reduction: *SAINT*; program(s) used to solve structure: *SHELXS97* (Sheldrick, 2008 ▶); program(s) used to refine structure: *SHELXL97* (Sheldrick, 2008 ▶); molecular graphics: *ORTEP-3* (Farrugia, 2012 ▶); software used to prepare material for publication: *SHELXL97*.

## Supplementary Material

Crystal structure: contains datablock(s) I, global. DOI: 10.1107/S1600536813001591/hb7027sup1.cif

Structure factors: contains datablock(s) I. DOI: 10.1107/S1600536813001591/hb7027Isup2.hkl

Supplementary material file. DOI: 10.1107/S1600536813001591/hb7027Isup3.cml

Additional supplementary materials:  crystallographic information; 3D view; checkCIF report

## Figures and Tables

**Table 1:** Hydrogen-bond geometry (Å, °) *Cg*3 is the centroid of the C15–C20 ring.

*D*—H⋯*A*	*D*—H	H⋯*A*	*D*⋯*A*	*D*—H⋯*A*
C6—H6⋯O1^i^	0.93	2.53	3.446 (3)	170
C11—H11⋯N2^ii^	0.93	2.59	3.435 (3)	152
C16—H16⋯O1^iii^	0.93	2.54	3.465 (3)	177
C1—H1*B*⋯*Cg*3^iv^	0.96	2.82	3.626 (3)	142

Symmetry codes: (i) 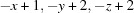; (ii) 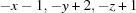; (iii) 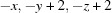; (iv) 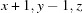.

**Fig. 1.**
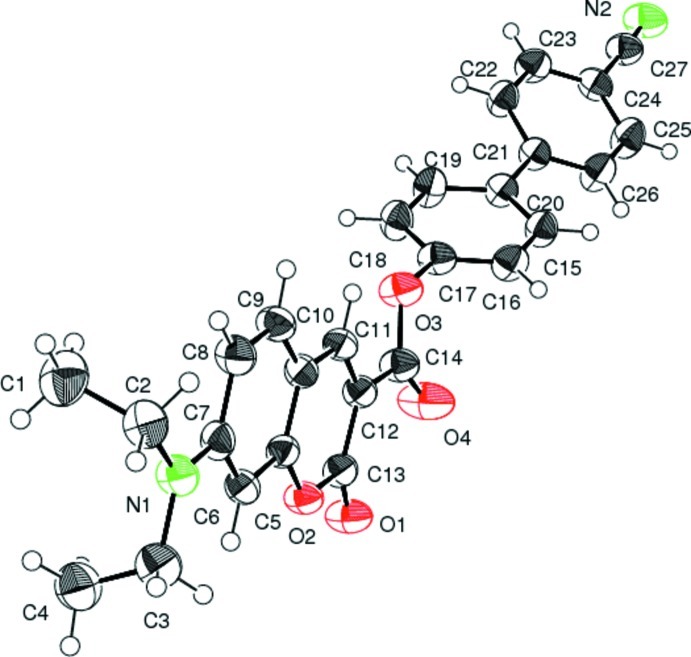
The molecular structure of the title compound. Displacement ellipsoids are drawn at the 50% probability level.

**Fig. 2.**
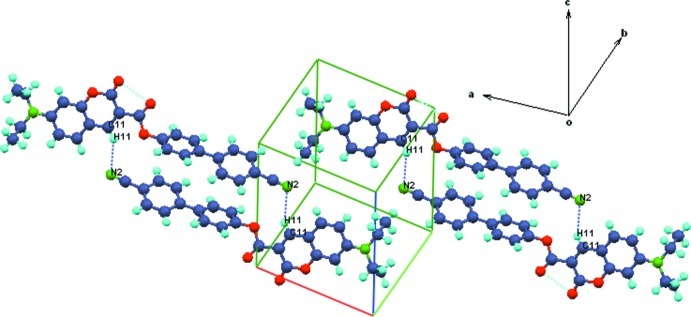
Crystal packing for the title compound with hydrogen bonds drawn as dashed lines.

## supplementary crystallographic information

### Comment

Coumarins are well known for displaying a broad range of biological activities like anti- inflammatory, anticonvulsant (Bhat *et al.*, 2006) and antitumor agents (Chimichi *et al.*, 2002). As part of our studies in this area, we now report the synthesis and crystal structure (Fig. 1) of the title cyanobiphenyl coumarin, (I), derived from the reaction of 7-diethylamino coumarin.

The 2*H*-chromene ring (O2/C5–C13) system in (I) is almost planar, with a maximum deviation of 0.025 (2) Å for atom C13. The dihedral angle between 2*H*-chromene ring (O2/C5–C13) with the benzene(C15–C20) terminal benzene(C21—C26) rings are 75.59 (8)° and 56.02 (1)° respectively. The crystal structure is characterized by intermolecular C6—H6···O1, C11—H11···N2 and C16—H16···O1 hydrogen bonding and also features C—H..π [C_g_(3)(C15–C20) interactions (Table.1). Crystal packing for the title compound with hydrogen bonds drawn as dashed lines (Fig. 2).

### Experimental

*N*,*N*-Dicyclohexylcarbodiimide (215 mg, 1.2 mmol) was added to a magnetically stirred solution of 7-(diethylamino) -2-oxo-2*H*-chromene -3-carboxylic acid (261 mg, 1 mmol), 4-cyano 4^'^-hydroxybiphenyl (195 mg, 1 mmol) and a catalytic quantity of *N*,*N*-dimethylaminopyrimidine (DMAP) in dried dichloromethane. Resultant reaction mixture was stirred further for 24 h at room temperature. Pure compound was obtained by aqueous workup and column chromatography. Colourless prisms were obtained from chloroform solution on slow evaporation at room temperature (m.p. 541 K).

### Refinement

All H atoms were positioned geometrically, with C—H = 0.93 Å for aromatic H, C—H = 0.97 Å for methylene H and C—H = 0.96 Å for methyl H,and refined using a riding model with *U*_iso_(H) = 1.5*U*_eq_(C) for methyl H and *U*_iso_(H) = 1.2*U*_eq_(C) for all other H.

### Crystal data

**Table:**

C_27_H_22_N_2_O_4_	*Z* = 2
*M**_r_* = 438.47	*F*(000) = 460
Triclinic, *P*1	*D*_x_ = 1.334 Mg m^−^^3^
Hall symbol: -P 1	Melting point: 541 K
*a* = 9.652 (3) Å	Mo *K*α radiation, λ = 0.71073 Å
*b* = 10.252 (4) Å	Cell parameters from 3783 reflections
*c* = 11.121 (4) Å	θ = 1.8–25.0°
α = 87.214 (10)°	µ = 0.09 mm^−^^1^
β = 86.358 (10)°	*T* = 300 K
γ = 84.348 (11)°	Prism, colourless
*V* = 1091.9 (6) Å^3^	0.24 × 0.20 × 0.18 mm

### Data collection

**Table:**

Bruker SMART CCD diffractometer	3783 independent reflections
Radiation source: fine-focus sealed tube	2495 reflections with *I* > 2σ(*I*)
Graphite monochromator	*R*_int_ = 0.048
ω and φ scans	θ_max_ = 25.0°, θ_min_ = 1.8°
Absorption correction: multi-scan (*SADABS*; Bruker, 2001)	*h* = −11→10
*T*_min_ = 0.979, *T*_max_ = 0.984	*k* = −12→12
10667 measured reflections	*l* = −13→13

### Refinement

**Table:**

Refinement on *F*^2^	Secondary atom site location: difference Fourier map
Least-squares matrix: full	Hydrogen site location: inferred from neighbouring sites
*R*[*F*^2^ > 2σ(*F*^2^)] = 0.058	H-atom parameters constrained
*wR*(*F*^2^) = 0.173	*w* = 1/[σ^2^(*F*_o_^2^) + (0.1107*P*)^2^] where *P* = (*F*_o_^2^ + 2*F*_c_^2^)/3
*S* = 0.97	(Δ/σ)_max_ < 0.001
3783 reflections	Δρ_max_ = 0.22 e Å^−^^3^
301 parameters	Δρ_min_ = −0.37 e Å^−^^3^
0 restraints	Extinction correction: *SHELXL97* (Sheldrick, 2008), Fc^*^=kFc[1+0.001xFc^2^λ^3^/sin(2θ)]^-1/4^
Primary atom site location: structure-invariant direct methods	Extinction coefficient: 0.014 (4)

### Special details

**Table:**

Experimental. ^1^H NMR (400Mz, TMS): δ 8.36(s, 1H), 7.44–7.65(m, 6H), 7.12–7.08(m, 3H), 6.34(m, 2H), 3.38(m, 4H), 1.10(m, 6H); Elemental analysis calculated for C_27_H_22_N_2_O_4_, C, 73.96; H, 5.06; N, 6.39; found C, 74.20; H, 5.32; N, 6.33.
Geometry. All s.u.'s (except the s.u. in the dihedral angle between two l.s. planes) are estimated using the full covariance matrix. The cell s.u.'s are taken into account individually in the estimation of s.u.'s in distances, angles and torsion angles; correlations between s.u.'s in cell parameters are only used when they are defined by crystal symmetry. An approximate (isotropic) treatment of cell s.u.'s is used for estimating s.u.'s involving l.s. planes.
Refinement. Refinement of *F*^2^ against ALL reflections. The weighted *R*-factor *wR* and goodness of fit *S* are based on *F*^2^, conventional *R*-factors *R* are based on *F*, with *F* set to zero for negative *F*^2^. The threshold expression of *F*^2^ > 2σ(*F*^2^) is used only for calculating *R*-factors(gt) *etc*. and is not relevant to the choice of reflections for refinement. *R*-factors based on *F*^2^ are statistically about twice as large as those based on *F*, and *R*- factors based on ALL data will be even larger.

### Fractional atomic coordinates and isotropic or equivalent isotropic displacement parameters (Å^2^)

**Table:**

	*x*	*y*	*z*	*U*_iso_*/*U*_eq_	
O1	0.24765 (18)	1.10054 (17)	0.94063 (14)	0.0535 (5)	
O2	0.37525 (15)	0.91308 (15)	0.92731 (12)	0.0417 (4)	
O3	−0.04165 (16)	0.96402 (16)	0.71953 (13)	0.0503 (5)	
O4	−0.0195 (2)	1.1161 (2)	0.85320 (18)	0.0753 (6)	
N1	0.68882 (19)	0.52702 (19)	0.90485 (16)	0.0457 (5)	
N2	−1.0656 (2)	1.3764 (2)	0.4192 (2)	0.0641 (7)	
C1	0.7967 (3)	0.4011 (3)	0.7316 (2)	0.0668 (8)	
H1A	0.7313	0.4453	0.6782	0.100*	
H1B	0.8213	0.3130	0.7068	0.100*	
H1C	0.8789	0.4469	0.7291	0.100*	
C2	0.7317 (3)	0.3978 (2)	0.8581 (2)	0.0493 (6)	
H2A	0.6507	0.3485	0.8602	0.059*	
H2B	0.7980	0.3515	0.9109	0.059*	
C3	0.7847 (3)	0.5811 (3)	0.9826 (2)	0.0529 (7)	
H3A	0.8333	0.5094	1.0280	0.064*	
H3B	0.7308	0.6370	1.0399	0.064*	
C4	0.8898 (3)	0.6584 (3)	0.9148 (2)	0.0656 (8)	
H4A	0.9469	0.6026	0.8608	0.098*	
H4B	0.9474	0.6927	0.9706	0.098*	
H4C	0.8426	0.7296	0.8695	0.098*	
C5	0.4066 (2)	0.7908 (2)	0.88368 (16)	0.0342 (5)	
C6	0.5309 (2)	0.7249 (2)	0.91367 (18)	0.0393 (6)	
H6	0.5901	0.7641	0.9607	0.047*	
C7	0.5690 (2)	0.5977 (2)	0.87307 (18)	0.0383 (5)	
C8	0.4763 (2)	0.5447 (2)	0.7979 (2)	0.0448 (6)	
H8	0.4998	0.4617	0.7681	0.054*	
C9	0.3544 (2)	0.6131 (2)	0.76906 (19)	0.0425 (6)	
H9	0.2965	0.5757	0.7195	0.051*	
C10	0.3130 (2)	0.7385 (2)	0.81176 (17)	0.0361 (5)	
C11	0.1893 (2)	0.8156 (2)	0.78679 (18)	0.0376 (5)	
H11	0.1267	0.7824	0.7388	0.045*	
C12	0.1576 (2)	0.9373 (2)	0.83021 (17)	0.0366 (5)	
C13	0.2546 (2)	0.9931 (2)	0.90192 (18)	0.0380 (5)	
C14	0.0264 (2)	1.0177 (2)	0.80661 (19)	0.0449 (6)	
C15	−0.4115 (2)	1.0844 (2)	0.73193 (18)	0.0441 (6)	
H15	−0.4890	1.0853	0.7861	0.053*	
C16	−0.2848 (2)	1.0282 (2)	0.76805 (18)	0.0451 (6)	
H16	−0.2770	0.9911	0.8456	0.054*	
C17	−0.1706 (2)	1.0274 (2)	0.68882 (19)	0.0416 (6)	
C18	−0.1806 (2)	1.0826 (2)	0.5739 (2)	0.0481 (6)	
H18	−0.1022	1.0822	0.5208	0.058*	
C19	−0.3081 (2)	1.1384 (3)	0.53862 (19)	0.0483 (6)	
H19	−0.3147	1.1757	0.4610	0.058*	
C20	−0.4266 (2)	1.1403 (2)	0.61565 (18)	0.0392 (6)	
C21	−0.5644 (2)	1.1962 (2)	0.57555 (17)	0.0384 (5)	
C22	−0.5909 (2)	1.2058 (2)	0.45324 (19)	0.0473 (6)	
H22	−0.5202	1.1791	0.3964	0.057*	
C23	−0.7190 (3)	1.2539 (3)	0.4155 (2)	0.0490 (6)	
H23	−0.7338	1.2605	0.3336	0.059*	
C24	−0.8261 (2)	1.2927 (2)	0.4976 (2)	0.0442 (6)	
C25	−0.8024 (3)	1.2853 (3)	0.6196 (2)	0.0567 (7)	
H25	−0.8733	1.3124	0.6761	0.068*	
C26	−0.6725 (3)	1.2373 (3)	0.6565 (2)	0.0534 (7)	
H26	−0.6573	1.2325	0.7384	0.064*	
C27	−0.9608 (3)	1.3402 (2)	0.4561 (2)	0.0490 (6)	

### Atomic displacement parameters (Å^2^)

**Table:**

	*U*^11^	*U*^22^	*U*^33^	*U*^12^	*U*^13^	*U*^23^
O1	0.0521 (11)	0.0481 (12)	0.0611 (10)	0.0095 (8)	−0.0207 (8)	−0.0165 (8)
O2	0.0338 (9)	0.0459 (10)	0.0452 (8)	0.0071 (7)	−0.0118 (7)	−0.0110 (7)
O3	0.0396 (10)	0.0559 (11)	0.0541 (9)	0.0177 (8)	−0.0193 (8)	−0.0138 (7)
O4	0.0577 (13)	0.0671 (14)	0.1019 (15)	0.0276 (10)	−0.0357 (11)	−0.0404 (11)
N1	0.0342 (12)	0.0484 (13)	0.0530 (11)	0.0099 (9)	−0.0095 (9)	−0.0056 (9)
N2	0.0480 (14)	0.0701 (17)	0.0754 (14)	0.0078 (12)	−0.0270 (12)	−0.0109 (12)
C1	0.068 (2)	0.0506 (18)	0.0788 (18)	0.0061 (14)	0.0090 (15)	−0.0119 (13)
C2	0.0434 (15)	0.0425 (15)	0.0594 (14)	0.0091 (12)	−0.0053 (11)	0.0016 (11)
C3	0.0398 (15)	0.0597 (18)	0.0569 (13)	0.0145 (12)	−0.0136 (11)	−0.0019 (12)
C4	0.0493 (18)	0.076 (2)	0.0709 (17)	0.0032 (15)	−0.0055 (13)	−0.0118 (14)
C5	0.0326 (13)	0.0376 (13)	0.0321 (10)	−0.0006 (10)	−0.0022 (9)	−0.0031 (8)
C6	0.0272 (13)	0.0503 (15)	0.0402 (11)	0.0010 (10)	−0.0060 (9)	−0.0051 (10)
C7	0.0288 (12)	0.0451 (15)	0.0392 (11)	0.0047 (10)	−0.0013 (9)	0.0000 (9)
C8	0.0362 (14)	0.0435 (15)	0.0537 (12)	0.0052 (11)	−0.0062 (10)	−0.0079 (10)
C9	0.0345 (14)	0.0462 (15)	0.0476 (12)	−0.0006 (11)	−0.0094 (10)	−0.0076 (10)
C10	0.0296 (13)	0.0418 (14)	0.0364 (10)	0.0004 (10)	−0.0047 (9)	−0.0019 (9)
C11	0.0309 (13)	0.0460 (15)	0.0363 (10)	−0.0018 (10)	−0.0079 (9)	−0.0019 (9)
C12	0.0319 (13)	0.0412 (14)	0.0361 (10)	0.0022 (10)	−0.0066 (9)	−0.0022 (9)
C13	0.0344 (13)	0.0426 (15)	0.0361 (10)	0.0045 (10)	−0.0054 (9)	−0.0042 (9)
C14	0.0419 (15)	0.0454 (16)	0.0473 (12)	0.0046 (12)	−0.0115 (11)	−0.0078 (11)
C15	0.0358 (14)	0.0542 (16)	0.0403 (11)	0.0034 (11)	−0.0019 (10)	0.0026 (10)
C16	0.0452 (16)	0.0518 (16)	0.0366 (11)	0.0054 (11)	−0.0093 (11)	0.0044 (10)
C17	0.0361 (14)	0.0406 (14)	0.0475 (12)	0.0081 (10)	−0.0137 (10)	−0.0053 (9)
C18	0.0355 (14)	0.0567 (17)	0.0491 (13)	0.0081 (12)	0.0002 (10)	0.0014 (11)
C19	0.0398 (15)	0.0617 (17)	0.0394 (11)	0.0094 (12)	−0.0030 (10)	0.0090 (10)
C20	0.0361 (13)	0.0407 (14)	0.0394 (11)	0.0044 (10)	−0.0050 (10)	0.0004 (9)
C21	0.0341 (13)	0.0407 (14)	0.0389 (11)	0.0039 (10)	−0.0047 (9)	0.0005 (9)
C22	0.0365 (14)	0.0634 (17)	0.0392 (11)	0.0073 (12)	−0.0019 (10)	−0.0004 (10)
C23	0.0471 (16)	0.0574 (17)	0.0420 (12)	0.0027 (12)	−0.0122 (11)	0.0013 (10)
C24	0.0322 (14)	0.0456 (15)	0.0544 (13)	0.0025 (11)	−0.0115 (11)	0.0003 (10)
C25	0.0391 (15)	0.078 (2)	0.0477 (13)	0.0180 (13)	−0.0009 (11)	−0.0029 (12)
C26	0.0428 (16)	0.0751 (19)	0.0386 (12)	0.0145 (13)	−0.0057 (11)	−0.0015 (11)
C27	0.0457 (16)	0.0495 (16)	0.0522 (13)	0.0011 (12)	−0.0120 (12)	−0.0058 (11)

### Geometric parameters (Å, º)

**Table:**

O1—C13	1.196 (3)	C9—C10	1.404 (3)
O2—C5	1.365 (3)	C9—H9	0.9300
O2—C13	1.393 (3)	C10—C11	1.401 (3)
O3—C14	1.370 (3)	C11—C12	1.360 (3)
O3—C17	1.400 (3)	C11—H11	0.9300
O4—C14	1.189 (3)	C12—C13	1.443 (3)
N1—C7	1.360 (3)	C12—C14	1.473 (3)
N1—C2	1.459 (3)	C15—C16	1.374 (3)
N1—C3	1.470 (3)	C15—C20	1.399 (3)
N2—C27	1.138 (3)	C15—H15	0.9300
C1—C2	1.504 (4)	C16—C17	1.367 (3)
C1—H1A	0.9600	C16—H16	0.9300
C1—H1B	0.9600	C17—C18	1.378 (3)
C1—H1C	0.9600	C18—C19	1.377 (3)
C2—H2A	0.9700	C18—H18	0.9300
C2—H2B	0.9700	C19—C20	1.384 (3)
C3—C4	1.493 (4)	C19—H19	0.9300
C3—H3A	0.9700	C20—C21	1.482 (3)
C3—H3B	0.9700	C21—C26	1.381 (3)
C4—H4A	0.9600	C21—C22	1.397 (3)
C4—H4B	0.9600	C22—C23	1.368 (3)
C4—H4C	0.9600	C22—H22	0.9300
C5—C6	1.370 (3)	C23—C24	1.377 (3)
C5—C10	1.405 (3)	C23—H23	0.9300
C6—C7	1.408 (3)	C24—C25	1.388 (3)
C6—H6	0.9300	C24—C27	1.439 (3)
C7—C8	1.424 (3)	C25—C26	1.380 (3)
C8—C9	1.358 (3)	C25—H25	0.9300
C8—H8	0.9300	C26—H26	0.9300
			
C5—O2—C13	123.43 (16)	C12—C11—H11	118.9
C14—O3—C17	117.47 (18)	C10—C11—H11	118.9
C7—N1—C2	121.64 (19)	C11—C12—C13	120.2 (2)
C7—N1—C3	121.2 (2)	C11—C12—C14	122.59 (19)
C2—N1—C3	117.10 (19)	C13—C12—C14	117.3 (2)
C2—C1—H1A	109.5	O1—C13—O2	114.84 (18)
C2—C1—H1B	109.5	O1—C13—C12	128.9 (2)
H1A—C1—H1B	109.5	O2—C13—C12	116.2 (2)
C2—C1—H1C	109.5	O4—C14—O3	122.0 (2)
H1A—C1—H1C	109.5	O4—C14—C12	127.6 (2)
H1B—C1—H1C	109.5	O3—C14—C12	110.4 (2)
N1—C2—C1	114.2 (2)	C16—C15—C20	121.6 (2)
N1—C2—H2A	108.7	C16—C15—H15	119.2
C1—C2—H2A	108.7	C20—C15—H15	119.2
N1—C2—H2B	108.7	C17—C16—C15	119.31 (18)
C1—C2—H2B	108.7	C17—C16—H16	120.3
H2A—C2—H2B	107.6	C15—C16—H16	120.3
N1—C3—C4	113.6 (2)	C16—C17—C18	121.0 (2)
N1—C3—H3A	108.8	C16—C17—O3	121.06 (18)
C4—C3—H3A	108.8	C18—C17—O3	117.9 (2)
N1—C3—H3B	108.8	C19—C18—C17	119.2 (2)
C4—C3—H3B	108.8	C19—C18—H18	120.4
H3A—C3—H3B	107.7	C17—C18—H18	120.4
C3—C4—H4A	109.5	C18—C19—C20	121.68 (19)
C3—C4—H4B	109.5	C18—C19—H19	119.2
H4A—C4—H4B	109.5	C20—C19—H19	119.2
C3—C4—H4C	109.5	C19—C20—C15	117.2 (2)
H4A—C4—H4C	109.5	C19—C20—C21	121.35 (18)
H4B—C4—H4C	109.5	C15—C20—C21	121.4 (2)
O2—C5—C6	116.87 (18)	C26—C21—C22	117.3 (2)
O2—C5—C10	119.93 (19)	C26—C21—C20	121.94 (18)
C6—C5—C10	123.2 (2)	C22—C21—C20	120.8 (2)
C5—C6—C7	119.85 (19)	C23—C22—C21	121.2 (2)
C5—C6—H6	120.1	C23—C22—H22	119.4
C7—C6—H6	120.1	C21—C22—H22	119.4
N1—C7—C6	121.63 (19)	C22—C23—C24	120.7 (2)
N1—C7—C8	120.9 (2)	C22—C23—H23	119.6
C6—C7—C8	117.5 (2)	C24—C23—H23	119.6
C9—C8—C7	121.2 (2)	C23—C24—C25	119.3 (2)
C9—C8—H8	119.4	C23—C24—C27	119.77 (19)
C7—C8—H8	119.4	C25—C24—C27	120.9 (2)
C8—C9—C10	122.0 (2)	C26—C25—C24	119.4 (2)
C8—C9—H9	119.0	C26—C25—H25	120.3
C10—C9—H9	119.0	C24—C25—H25	120.3
C11—C10—C9	125.81 (19)	C25—C26—C21	122.1 (2)
C11—C10—C5	117.9 (2)	C25—C26—H26	119.0
C9—C10—C5	116.2 (2)	C21—C26—H26	119.0
C12—C11—C10	122.29 (19)	N2—C27—C24	177.5 (3)
			
C7—N1—C2—C1	78.6 (3)	C17—O3—C14—C12	178.42 (18)
C3—N1—C2—C1	−98.1 (2)	C11—C12—C14—O4	168.8 (3)
C7—N1—C3—C4	−87.7 (3)	C13—C12—C14—O4	−11.6 (4)
C2—N1—C3—C4	88.9 (3)	C11—C12—C14—O3	−11.4 (3)
C13—O2—C5—C6	178.28 (18)	C13—C12—C14—O3	168.26 (18)
C13—O2—C5—C10	−1.8 (3)	C20—C15—C16—C17	0.4 (4)
O2—C5—C6—C7	179.15 (18)	C15—C16—C17—C18	0.3 (4)
C10—C5—C6—C7	−0.8 (3)	C15—C16—C17—O3	−176.0 (2)
C2—N1—C7—C6	−177.0 (2)	C14—O3—C17—C16	−68.9 (3)
C3—N1—C7—C6	−0.5 (3)	C14—O3—C17—C18	114.7 (2)
C2—N1—C7—C8	3.6 (3)	C16—C17—C18—C19	−0.5 (4)
C3—N1—C7—C8	−179.86 (19)	O3—C17—C18—C19	176.0 (2)
C5—C6—C7—N1	−177.34 (19)	C17—C18—C19—C20	−0.1 (4)
C5—C6—C7—C8	2.1 (3)	C18—C19—C20—C15	0.7 (4)
N1—C7—C8—C9	177.9 (2)	C18—C19—C20—C21	−177.7 (2)
C6—C7—C8—C9	−1.6 (3)	C16—C15—C20—C19	−0.9 (4)
C7—C8—C9—C10	−0.3 (3)	C16—C15—C20—C21	177.6 (2)
C8—C9—C10—C11	−179.9 (2)	C19—C20—C21—C26	−159.0 (2)
C8—C9—C10—C5	1.6 (3)	C15—C20—C21—C26	22.6 (4)
O2—C5—C10—C11	0.3 (3)	C19—C20—C21—C22	22.7 (3)
C6—C5—C10—C11	−179.71 (19)	C15—C20—C21—C22	−155.6 (2)
O2—C5—C10—C9	179.01 (18)	C26—C21—C22—C23	−0.1 (3)
C6—C5—C10—C9	−1.0 (3)	C20—C21—C22—C23	178.2 (2)
C9—C10—C11—C12	−179.0 (2)	C21—C22—C23—C24	−0.8 (4)
C5—C10—C11—C12	−0.4 (3)	C22—C23—C24—C25	1.4 (4)
C10—C11—C12—C13	1.8 (3)	C22—C23—C24—C27	−178.6 (2)
C10—C11—C12—C14	−178.57 (19)	C23—C24—C25—C26	−1.0 (4)
C5—O2—C13—O1	−175.22 (18)	C27—C24—C25—C26	179.0 (2)
C5—O2—C13—C12	3.0 (3)	C24—C25—C26—C21	0.1 (4)
C11—C12—C13—O1	175.0 (2)	C22—C21—C26—C25	0.5 (4)
C14—C12—C13—O1	−4.7 (3)	C20—C21—C26—C25	−177.8 (2)
C11—C12—C13—O2	−3.0 (3)	C23—C24—C27—N2	8 (6)
C14—C12—C13—O2	177.36 (17)	C25—C24—C27—N2	−172 (6)
C17—O3—C14—O4	−1.7 (3)		

### Hydrogen-bond geometry (Å, º)

Cg3 is the centroid of the C15–C20 ring.

**Table:**

*D*—H···*A*	*D*—H	H···*A*	*D*···*A*	*D*—H···*A*
C6—H6···O1^i^	0.93	2.53	3.446 (3)	170
C11—H11···N2^ii^	0.93	2.59	3.435 (3)	152
C16—H16···O1^iii^	0.93	2.54	3.465 (3)	177
C1—H1*B*···*Cg*3^iv^	0.96	2.82	3.626 (3)	142

Symmetry codes: (i) −*x*+1, −*y*+2, −*z*+2; (ii) −*x*−1, −*y*+2, −*z*+1; (iii) −*x*, −*y*+2, −*z*+2; (iv) *x*+1, *y*−1, *z*.
